# Supplementary material for: Lipidome Plasticity Enables Unusual Photosynthetic Flexibility in Arctic vs. Temperate Diatoms
Source: Mar Drugs. 2024 Jan 27;22(2):67. doi: 10.3390/md22020067 (PMC10890139; doi:10.3390/md22020067)
Supplement: Supplementary file 1 [file marinedrugs-22-00067-s001.zip › marinedrugs-2835868-supplementary.pdf]

Supplementary Materials

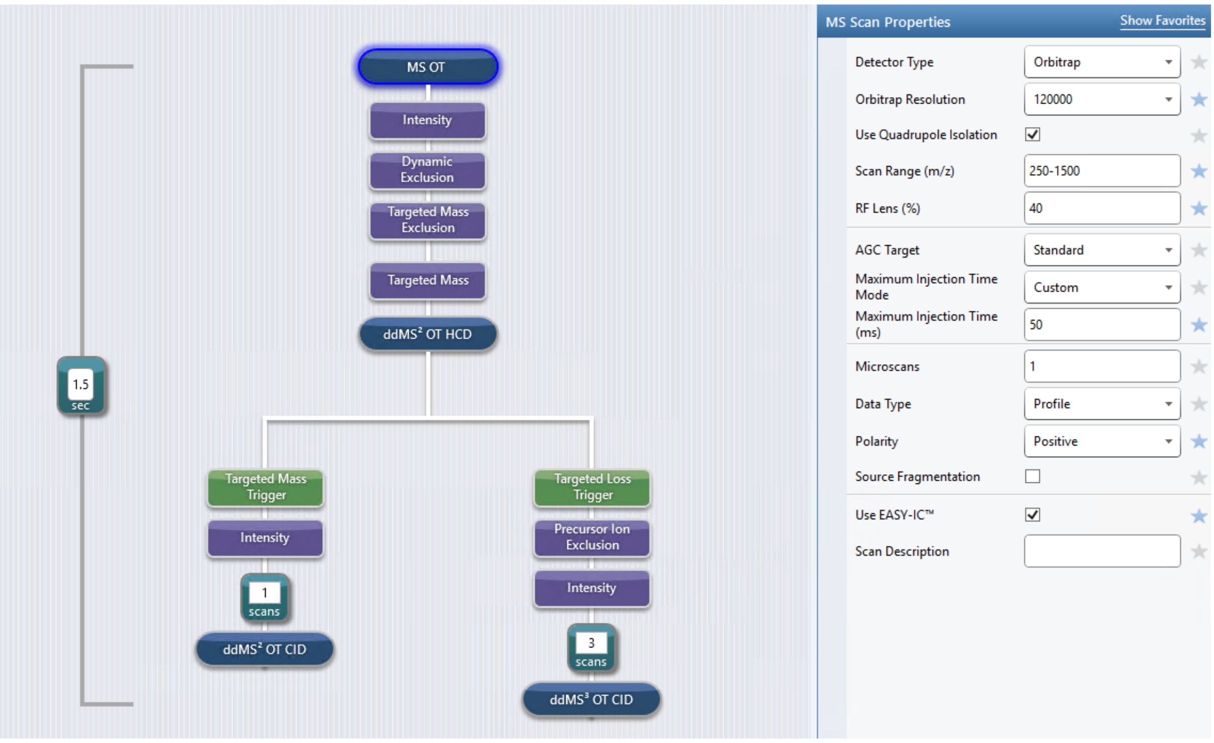

Figure S1: AcquireX DS DDA method – MS orbitrap workflow and masterscan configuration

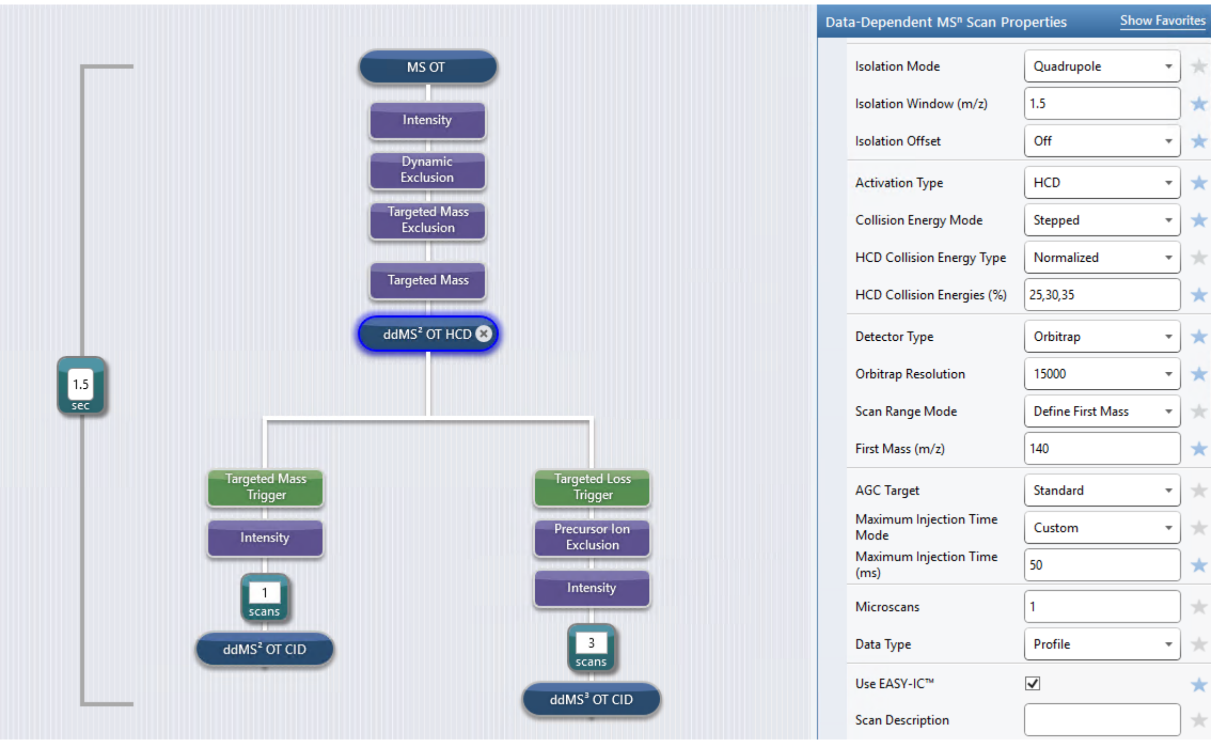

Figure S2: AcquireX DS DDA method – MS orbitrap workflow and ddMS2 HCD configurations

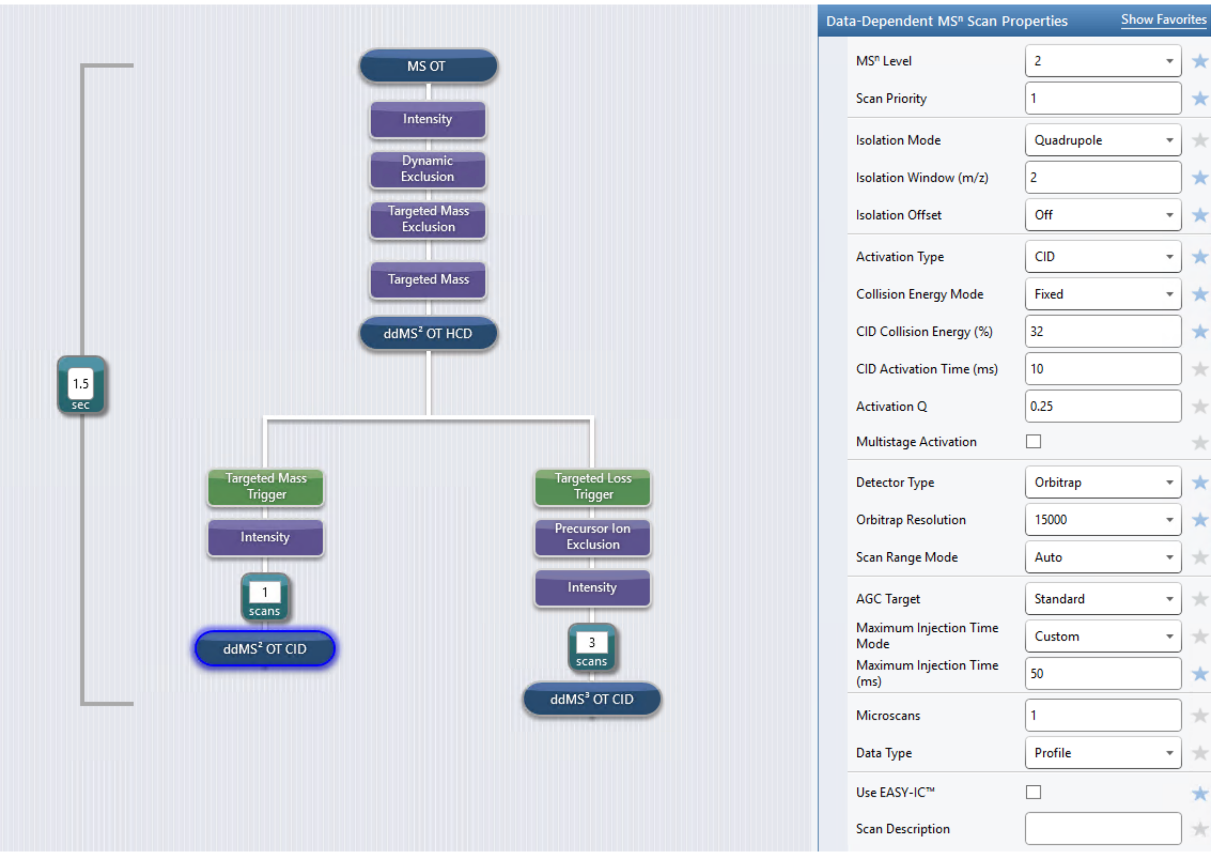

Figure S3: AcquireX DS DDA method – MS orbitrap workflow and ddMS2 CID configurations

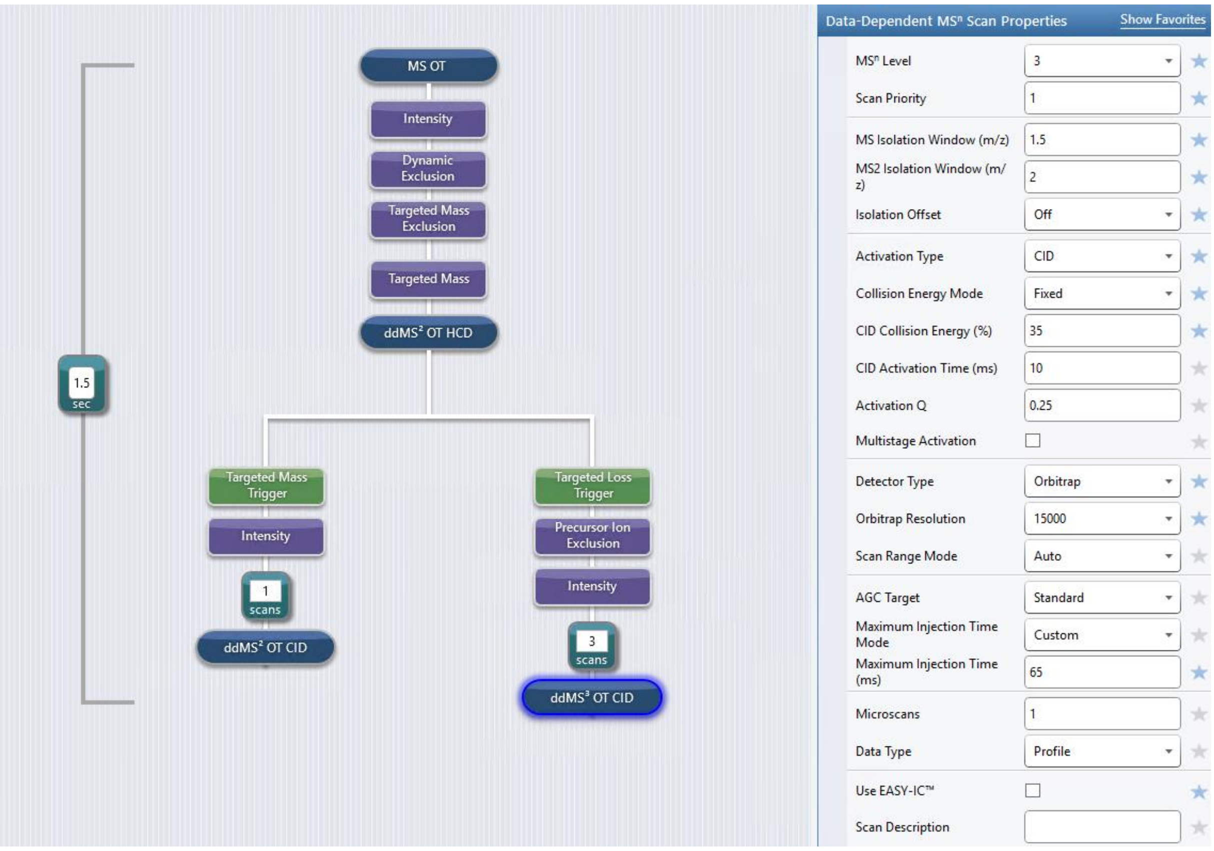

Figure S4: AcquireX DS DDA method – MS orbitrap workflow and ddMS3 HCD configurations

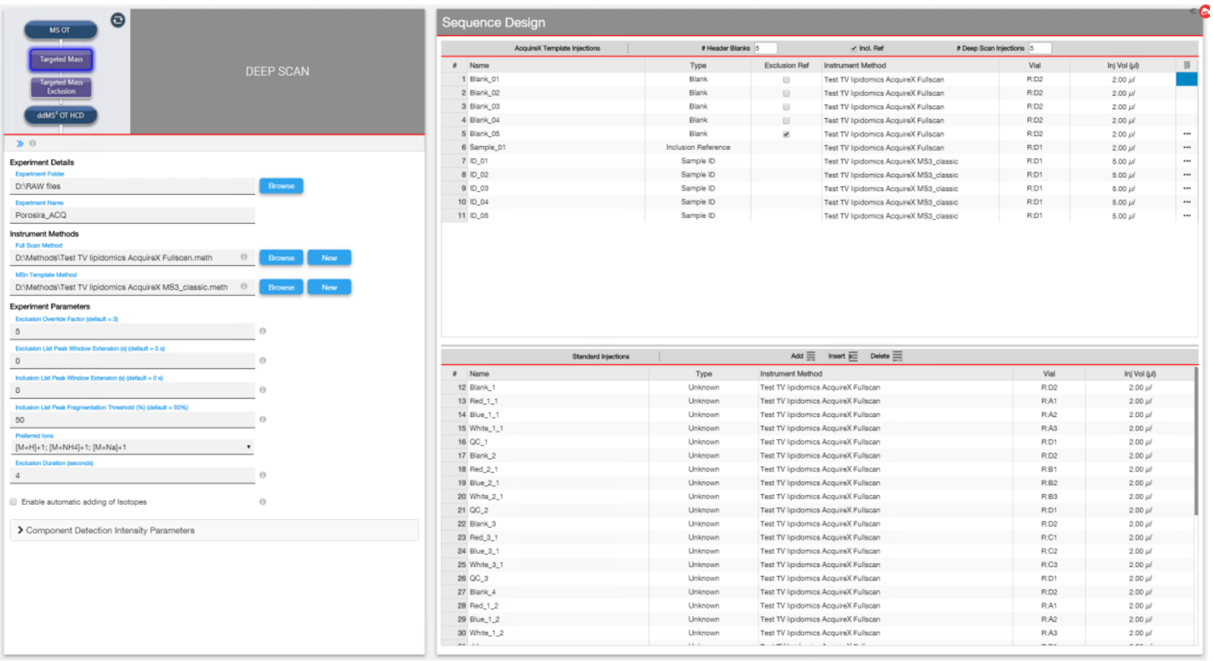

Figure S5: AcquireX DS DDA method – Experiment set up and injection sequence. Line 12-26 in injection sequence repeats 3 times.

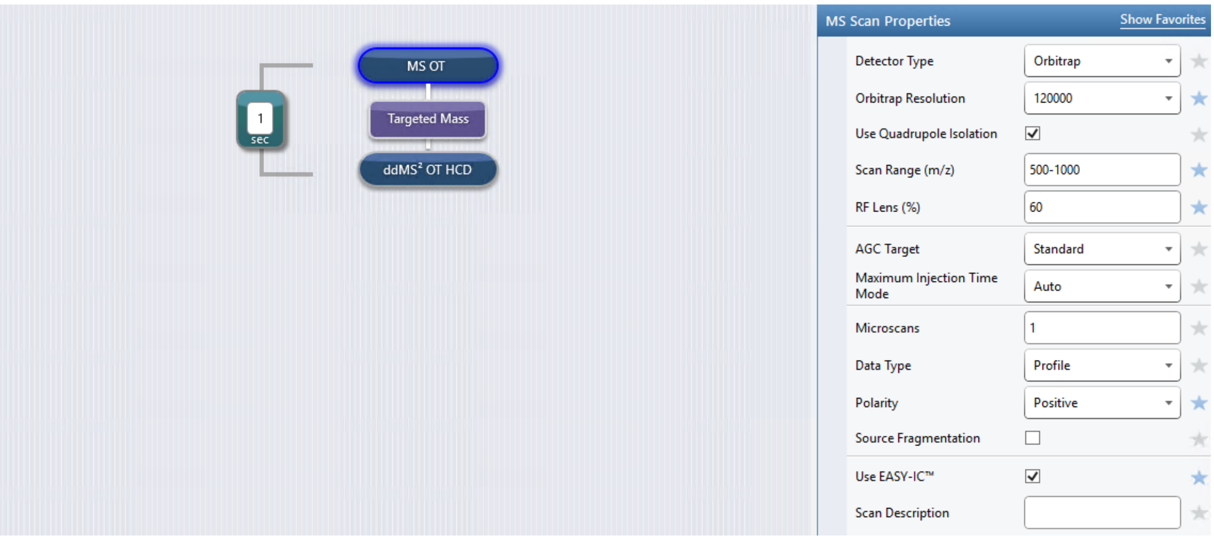

Figure S6: Targeted ddMS2 method for spectral library creation – masterscan configuration

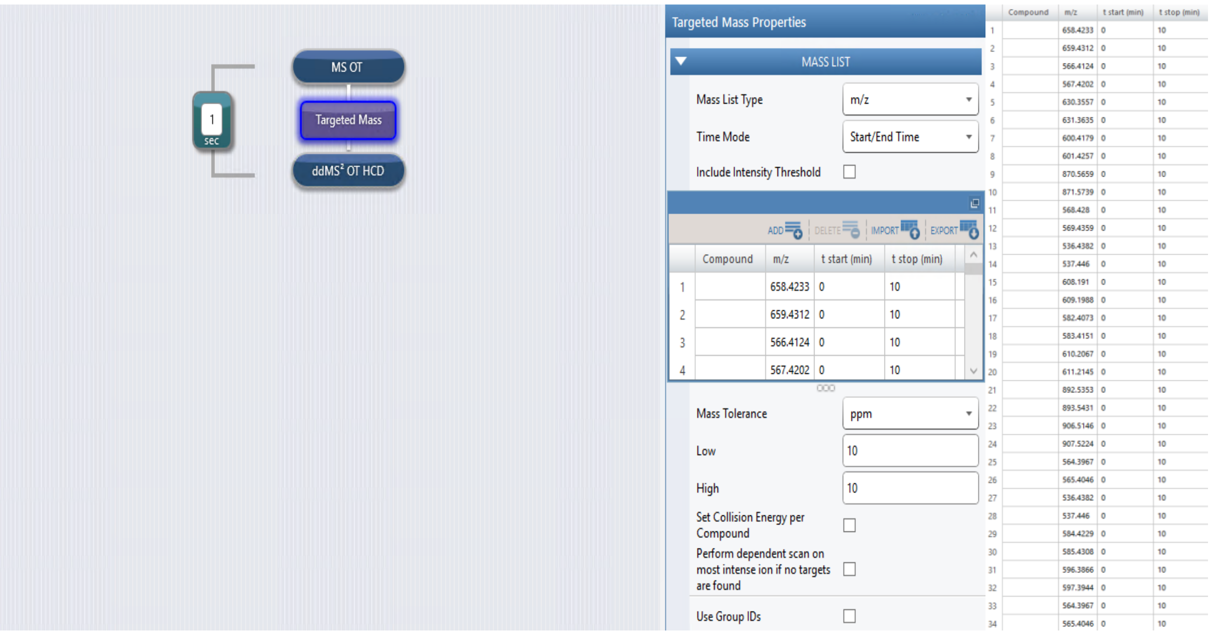

Figure S7: Targeted ddMS2 method for spectral library creation – Target mass list

Table S1: Mass list for pigment library creation

Compound	m/z
Fucoxanthin [M*]$^+$	658.4233
Fucoxanthin [M+H]$^+$	659.4312
Diatoxanthin [M*]$^+$	566.4124
Diatoxanthin [M+H]$^+$	567.4202
Peridinin [M*]$^+$	630.3557

Peridinin $[M+H]^+$	631.3635
Violaxanthin $[M*]^+$	600.4179
Violaxanthin $[M+H]^+$	601.4257
Pheophytin *a* $[M*]^+$	870.5659
Pheophytin *a* $[M+H]^+$	871.5739
Zeaxanthin $[M*]^+$	568.428
Zeaxanthin $[M+H]^+$	569.4359
Lycopene $[M*]^+$	536.4382
Lycopene $[M+H]^+$	537.446
Chlorophyll c_2 $[M*]^+$	608.191
Chlorophyll c_2 $[M+H]^+$	609.1988
Diadinoxanthin $[M*]^+$	582.4073
Diadinoxanthin $[M+H]^+$	583.4151
Chlorophyll c_1 $[M*]^+$	610.2067
Chlorophyll c_1 $[M+H]^+$	611.2145
Chlorophyll a $[M*]^+$	892.5353
Chlorophyll a $[M+H]^+$	893.5431
Chlorophyll b $[M*]^+$	906.5146
Chlorophyll b $[M+H]^+$	907.5224
Canthaxanthin $[M*]^+$	564.3967
Canthaxanthin $[M+H]^+$	565.4046
β-carotene $[M*]^+$	536.4382
β-carotene $[M+H]^+$	537.446

Antheraxanthin $[M^*]^+$	584.4229
Antheraxanthin $[M+H]^+$	585.4308
Astaxanthin $[M^*]^+$	596.3866
Astaxanthin $[M+H]^+$	597.3944
Alloxanthin $[M^*]^+$	564.3967
Alloxanthin $[M+H]^+$	565.4046

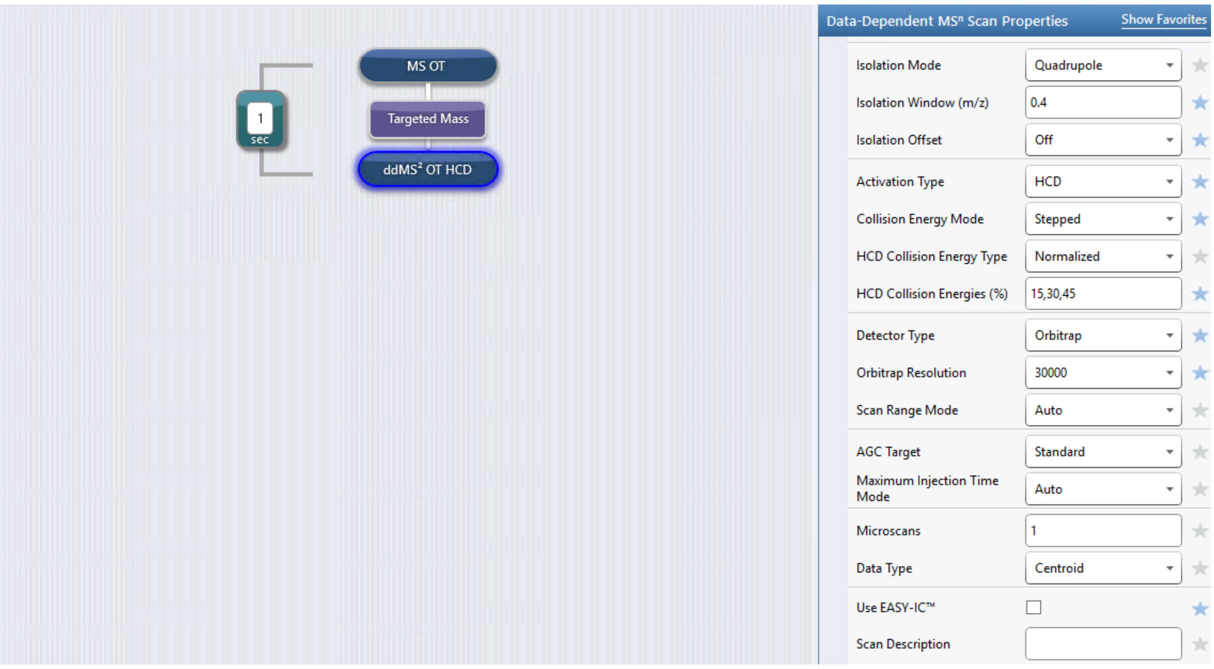

Figure S8: Targeted ddMS2 method for spectral library creation – MS orbitrap workflow and ddMS2 CID configurations

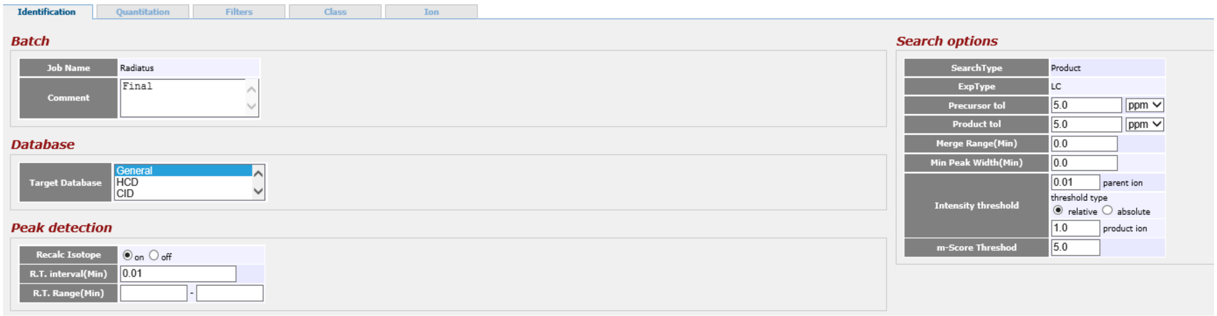

Figure S9: LipidSearch Identification configuration

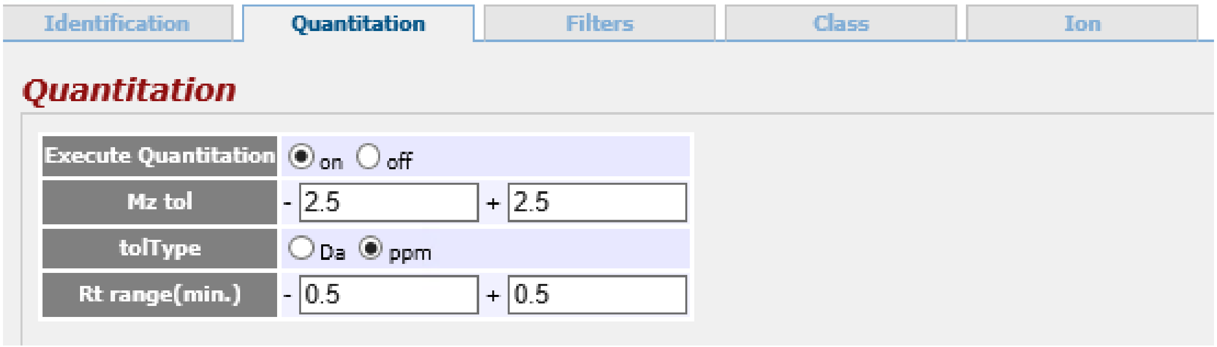

Figure S10: LipidSearch Quantitation configuration

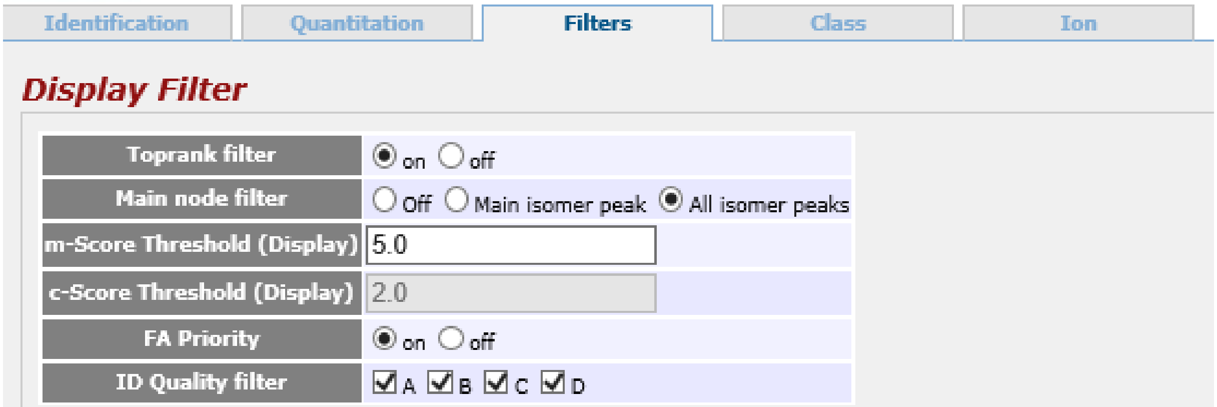

Figure S11: LipidSearch Filters configuration

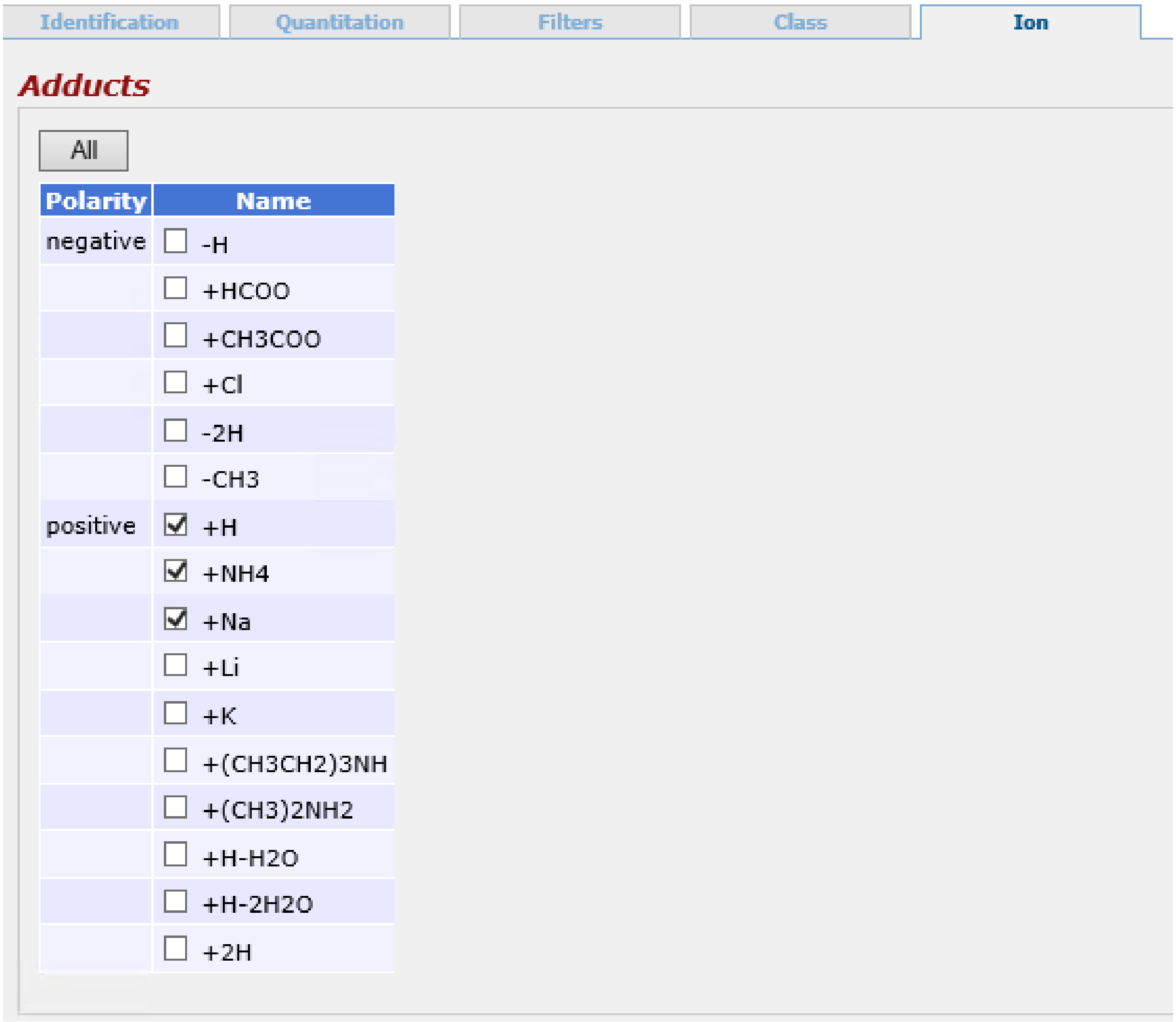

Figure S12: LipidSearch Adduct selection

Parameters

SearchType	Product
ExpType	LC
Normalize Type	None
Alignment Method	Mean
R.T. Tolerance	2.0
Calculate unassigned peak area	on
Filter Type	New Filter
Toprank Filter	on
Main Node Filter	All isomer peaks
m-Score Threshold	5.0
c-Score Threshold	2.0
ID Quality filter	[A, B, C, D]

Sample

c-1	Porosira_NY	TV_TM_201113_Rs_2.raw
c-2	Porosira_NY	TV_TM_201113_Rs_3.raw
c-3	Porosira_NY	TV_TM_201113_Rs_1.raw
s1-1	Porosira_NY	TV_TM_201113_R1_3.raw
s1-2	Porosira_NY	TV_TM_201113_R1_1.raw
s1-3	Porosira_NY	TV_TM_201113_R1_2.raw
s10-1	NY_ID	TV_TM_201213_ID_05.raw
s10-2	NY_ID	TV_TM_201213_ID_04.raw
s10-3	NY_ID	TV_TM_201213_ID_03.raw
s10-4	NY_ID	TV_TM_201213_ID_02.raw
s10-5	NY_ID	TV_TM_201213_ID_01.raw
s2-1	Porosira_NY	TV_TM_201113_R2_2.raw
s2-2	Porosira_NY	TV_TM_201113_R2_3.raw
s2-3	Porosira_NY	TV_TM_201113_R2_1.raw
s3-1	Porosira_NY	TV_TM_201113_R3_3.raw
s3-2	Porosira_NY	TV_TM_201113_R3_2.raw
s3-3	Porosira_NY	TV_TM_201113_R3_1.raw
s4-1	Porosira_NY	TV_TM_201113_B1_3.raw
s4-2	Porosira_NY	TV_TM_201113_B1_1.raw
s4-3	Porosira_NY	TV_TM_201113_B1_2.raw
s5-1	Porosira_NY	TV_TM_201113_B2_3.raw
s5-2	Porosira_NY	TV_TM_201113_B2_2.raw
s5-3	Porosira_NY	TV_TM_201113_B2_1.raw
s6-1	Porosira_NY	TV_TM_201113_B3_2.raw
s6-2	Porosira_NY	TV_TM_201113_B3_3.raw
s6-3	Porosira_NY	TV_TM_201113_B3_1.raw
s7-1	Porosira_NY	TV_TM_201113_W1_3.raw
s7-2	Porosira_NY	TV_TM_201113_W1_1.raw
s7-3	Porosira_NY	TV_TM_201113_W1_2.raw
s8-1	Porosira_NY	TV_TM_201113_W2_3.raw
s8-2	Porosira_NY	TV_TM_201113_W2_2.raw
s8-3	Porosira_NY	TV_TM_201113_W2_1.raw
s9-1	Porosira_NY	TV_TM_201113_W3_3.raw
s9-2	Porosira_NY	TV_TM_201113_W3_2.raw
s9-3	Porosira_NY	TV_TM_201113_W3_1.raw

Figure S13: Alignment settings in LipidSearch

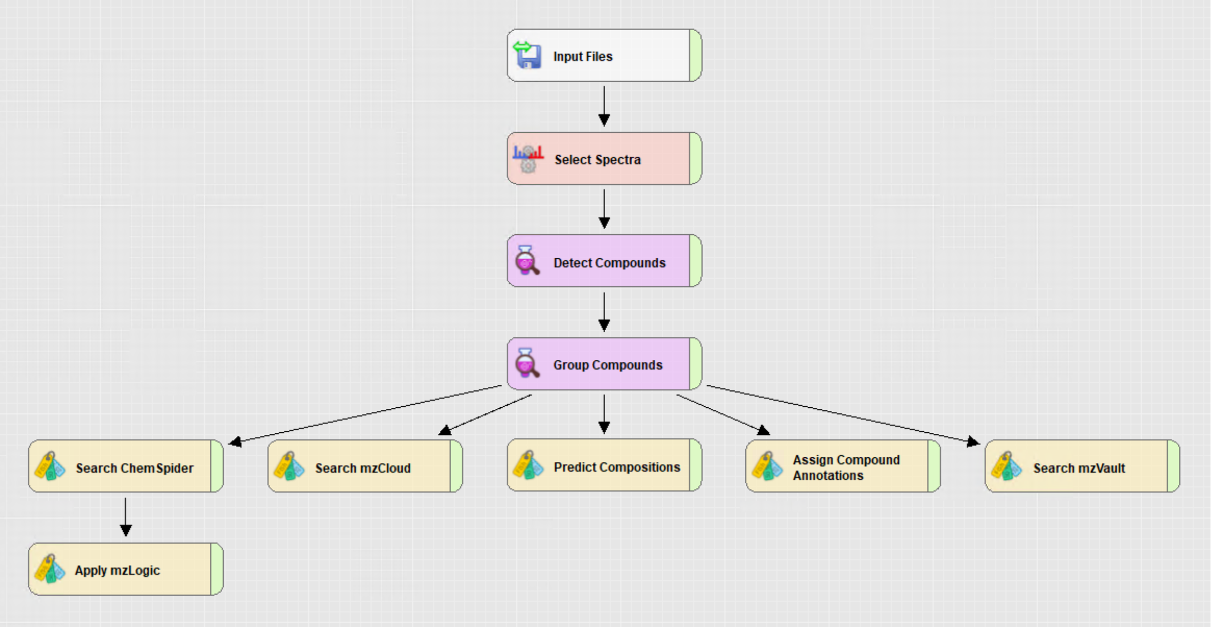

Figure S14: Compound discoverer workflow for data processing

Table S2: Compound Discoverer workflow parameter specifications

	**Targeted analysis**
**Select spectra**	
Lower RT limit	0 (lowest available RT is used)
Upper RT limit	0 (highest available RT is used)
Polarity	Any
**Detect Compounds**	
Mass tolerance	5 ppm
Min. Peak Intensity	250000
Use Most Intense Isotope Only	True
Chromatographic S/N threshold	1.5
Remove Baseline	False
Isotope Pattern Detection	Group Isotopes
Compound detection Ions	$[M+2H]^{2+}$, $[M+H]^{+}$
**Group Compounds**	
Mass tolerance	5 ppm
RT tolerance	0.2
Align Peaks	False
Preferred Ions	$[M+H-H2O]^{+}$, $[M+H]^{+}$
Areal Integration	Most Common Ion
Area Contribution	3
CV Contribution	10
FWHM to Base Contribution	5
Jaggedness Contribution	5
Modality Contribution	5
Zig-Zag Index Contribution	5
Peak Rating Threshold	4
Number of files	1

**Search ChemSpider**	
Database(s)	KEGG
Search Mode	By formula or mass
Mass Tolerance	5 ppm
Max. # of results per compound	100
Max. # of Predicted Compounds to be searched for per [illegible]	3
**Apply mzLogic**	
Max.# Compounds	0 (all candidates of all compounds are scored)
Max. #mzCloud Similarity Results to consider per [illegible]	10
Match Factor Threshold	30
**Search mzCloud**	
Compound Classes	All
Library	Autoprocessed; Reference
Search MSn Tree	False
Identity Search	HighChem High Res
Match Activation Type	True
Match Activation Energy	Match with Tolerance
Activation Energy Threshold	20
Apply Intensity Threshold	True
Similarity Search	None
Match Factor Threshold	60
Use DIA Scans for Search	False
Max. Isolation Width (DA)	500
Match Activation Type	False
Match Activation Energy	Any
Activation Energy Tolerance	100
Apply Intensity threshold	False
Match Factor Threshold	20
**Predict Compositions**	
Mass Tolerance	10 ppm
Min. Element Counts	C H
Max. Element Counts	C90 H190 Br3 Cl4 N10 O18 P3 S5
Min. RDBE	0
Max. RDBE	40
Min. H/C	0.1
Max. H/C	3.5
Max. # Candidates	10
Intensity Tolerance (%)	30
Intensity Threshold (%)	0.1
S/N Threshold	3
Use Dynamic Recalibration	True
Use Fragments Matching	True
Mass Tolerance	5 ppm
S/N Threshold	3
**Assign Compound Annotations**	
Mass Tolerance	5 ppm
Data Source #1	MzVault Search
Data Source #2	Predicted Compositions
Data Source #3	mzCloud Search

Data Source #4	ChemSpider Search
Data Source #5	-
Use mzLogic	True
Use Spectral Distance	True
SFit Threshold	20
SFit Range	20
Clear Names	False
**Search mzVault**	
mzVault Library	pigmentDB2.db
Compound Classes	All
Match Ion Activation Type	True
Match Ion Activation Energy	Match with Tolerance
Ion Activation Energy Tolerance	20
Match Ionization Method	True
Apply Intensity Threshold	True
Precursor Mass Tolerance	10 ppm
Match Analyzer Type	True
Search Algorithm	HighChem HighRes
Match Factor Threshold	50
RT Tolerance (min)	2
Use Retention Time	False
